# Stimulation within the cuneate nucleus suppresses synaptic activation of climbing fibers

**DOI:** 10.3389/fncir.2012.00120

**Published:** 2013-01-17

**Authors:** Pontus Geborek, Henrik Jörntell, Fredrik Bengtsson

**Affiliations:** Department of Experimental Medical Science, Lund UniversityLund, Sweden

**Keywords:** cuneate nucleus, inferior olive, inhibition, climbing fiber field response, pyramidal tract

## Abstract

Several lines of research have shown that the excitability of the inferior olive is suppressed during different phases of movement. A number of different structures like the cerebral cortex, the red nucleus, and the cerebellum have been suggested as candidate structures for mediating this gating. The inhibition of the responses of the inferior olivary neurons from the red nucleus has been studied extensively and anatomical studies have found specific areas within the cuneate nucleus to be target areas for projections from the magnocellular red nucleus. In addition, GABA-ergic cells projecting from the cuneate nucleus to the inferior olive have been found. We therefore tested if direct stimulation of the cuneate nucleus had inhibitory effects on a climbing fiber field response, evoked by electrical stimulation of the pyramidal tract, recorded on the surface of the cerebellum. When the pyramidal tract stimulation was preceded by weak electrical stimulation (5–20 μA) within the cuneate nucleus, the amplitude of the climbing fiber field potential was strongly suppressed (approx. 90% reduction). The time course of this suppression was similar to that found after red nucleus stimulation, with a peak suppression occurring at 70 ms after the cuneate stimulation. Application of CNQX (6-cyano-7-nitroquinoxaline-2,3-dione, disodium salt) on the cuneate nucleus blocked the suppression almost completely. We conclude that a relay through the cuneate nucleus is a possible pathway for movement-related suppression of climbing fiber excitability.

## Introduction

Essential to all theories of cerebellar function is the causes and conditions of climbing fiber activation. Several groups have shown that climbing fiber excitability is not constant but may change during different conditions in relation to movement. For example, the forelimb area of the C1-C3-Y zone system of the cerebellar cortex, which is innervated by the rostral subdivision of the dorsal accessory subdivision of the inferior olive (rDAO), is devoted to forelimb movement control via the motor cortex and the red nucleus. However, during different phases of movements, like reach-to-grasp (Gellman et al., 1985; Horn et al., 2004) and locomotion (Lidierth and Apps, 1990; Apps, 1999) climbing fiber excitability in the rDAO, is strongly modulated. Observations such as these have led to the idea of gating of synaptic transmission in the inferior olive (IO) during movement. An example of gating would be if an excitatory and an inhibitory synapse converged on the same cell, where the inhibitory synapse has the ability to prevent somatic depolarization from reaching firing threshold. An obvious candidate system for this gating would be the inhibitory nucleo-olivary pathway (Hesslow, 1986; Bengtsson and Hesslow, 2006). It has also been shown that stimulation of the magnocellular part of the red nucleus (RNm) inhibits responses evoked from the forelimb in rDAO neurons (Weiss et al., 1990; Horn et al., 1998; Gibson et al., 2002). This would represent a pathway by which the motor command itself can actively suppress climbing fiber discharge. This inhibition does not involve the cerebellum since the inhibition evoked from the red nucleus persisted after the cerebellum had been removed. Since the projection neurons of the RNm are not known to be inhibitory, RNm stimulation probably activates an additional pathway that has inhibitory effects in the IO.

**Figure 1:**
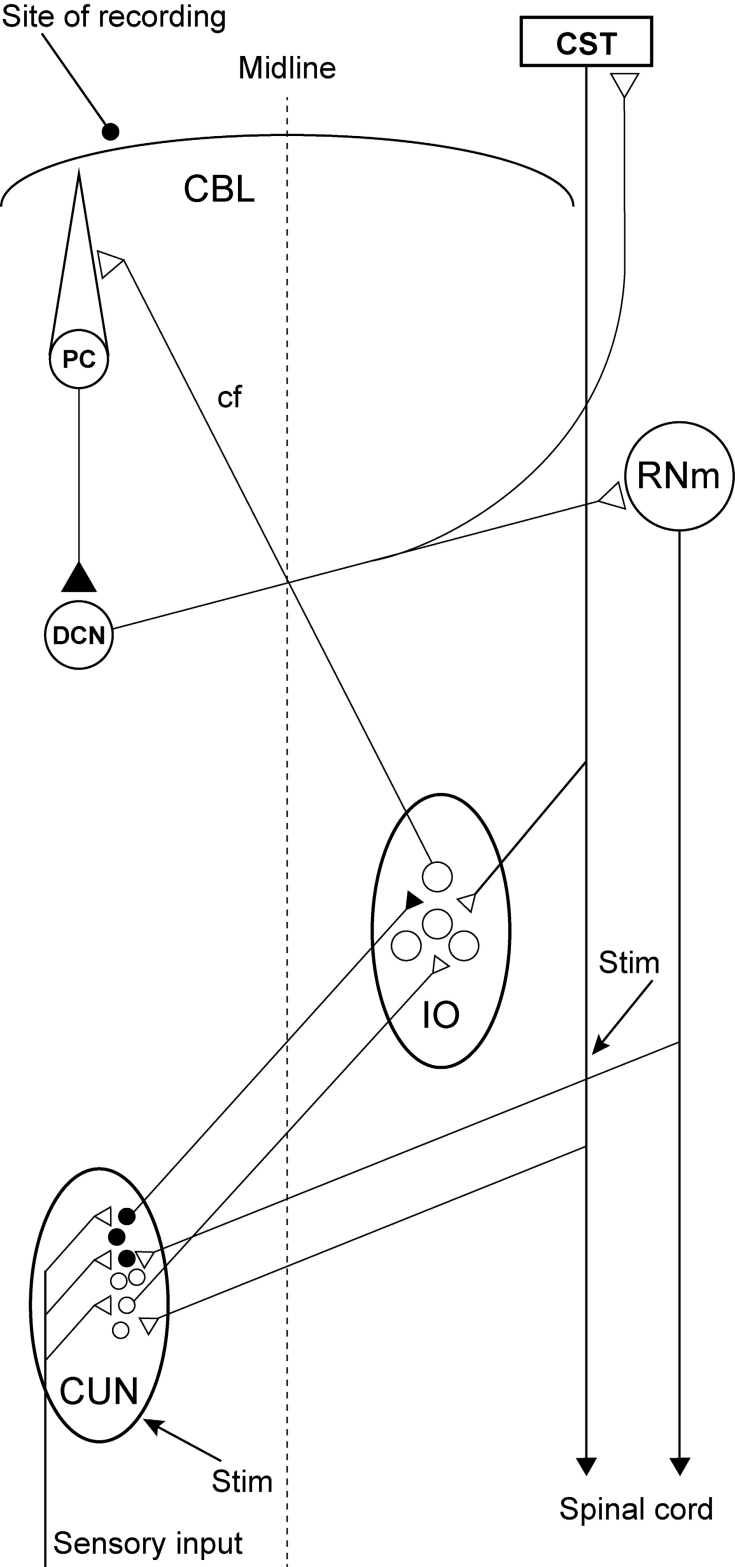
**Experimental set-up and schematic diagram outlining the anatomical connectivity of the structures studied.** RNm, Magno cellular red nucleus; IO, inferior olive; CUN, main cuneate; CBL, the cerebellum; PC, Purkinje cell; DCN, deep cerebellar nucleus; CST, cortico spinal tract. Inhibitory neurons (filled circles).

Although numerous investigations were carried out to determine the location of this inhibitory pathway or the structure that mediates this effect, its location is still unknown. However, a possible candidate has been identified: descending fibers from the red nucleus and the cerebral cortex converge in a specific region of the cuneate nucleus in which the neurons have efferent projections to the rDAO (Kuypers and Tuerk, 1964; Holstege and Kuypers, 1982; McCurdy et al., 1992, 1998). The purpose of the present study is to investigate directly if this region of the cuneate nucleus has inhibitory effects on inferior olivary neurons. For this purpose, we use microelectrodes to stimulate within the caudal part of the cuneate nucleus and test the inhibitory effects on synaptic activation of climbing fiber field potentials in the C3 zone of the cerebellar cortex (see Figure 1).

## Materials and methods

Seven adult cats were prepared as previously described (Ekerot and Jorntell, 2001; Jorntell and Ekerot, 2002, 2003). Briefly, the animals were initially anesthesia with propofol (Diprivan® Zeneca Ltd., Macclesfield Cheshire, UK), and mounted in a stereotaxic frame and decerebrated at a high decerebration point (just rostral to the superior colliculus). Subsequent to this, the anesthesia was terminated and the animals were kept paralyzed with pancuronium (Pavulon â Organon Teknika B.V., Boxtel, Holland) throughout the experiment. The animals were artificially ventilated and the end-expiratory CO_2_, blood pressure and rectal temperature were continuously monitored and maintained within physiological limits. Drainage of cerebrospinal fluid, pneumothorax and clamping the spinal processes of a few cervical and lumbar vertebral bodies served to increase the mechanical stability of the preparation. EEG recordings were characterized by a background of periodic 1–4 Hz oscillatory activity, periodically interrupted by large-amplitude 7–14 Hz spindle oscillations lasting for 0.5 s or more. These forms of EEG activities are normally associated with deep stages of sleep cf. (Niedermayer and Lopes Da Silva, 1993). The pattern of EEG activity and the blood pressure remained stable, also on noxious stimulation, throughout experiments.

### Recordings and stimulation

The initial delineation of the forelimb area of the C3 zone in the cerebellar anterior lobe was performed as described previously (Jorntell and Ekerot, 2003). In order to access the cuneate nucleus the occipital bone surrounding the foramen magnum was cut 7 mm rostrally. In addition, the bone of the first cervical vertebra was cut to the rostral border of the second cervical vertebra. Subsequently, a tungsten-in-glass microelectrode, with an exposed metal tip of 10–30 μm, was placed stereotaxically in the pyramidal tract just caudal (2 mm) to the IO at a depth of 4 mm from the surface of the brainstem, 1 mm lateral to the midline. The effectiveness of the pyramidal tract stimulation was verified by recording pyramidal tract volleys in the contralateral dorsolateral funiculus and, in some cases, through recordings from alpha-motoneurons that were synaptically excited by the pyramidal tract stimulation. The pyramidal tract stimulation was confirmed to evoke synaptic (in contrast to directly excited climbing fiber axons, cf. Jorntell and Ekerot, 2003) climbing fiber responses by recording with a silver ball electrode (Ø = 250 μm) placed on the surface in the C3 zone of the cerebellar cortex.

**Figure 2:**
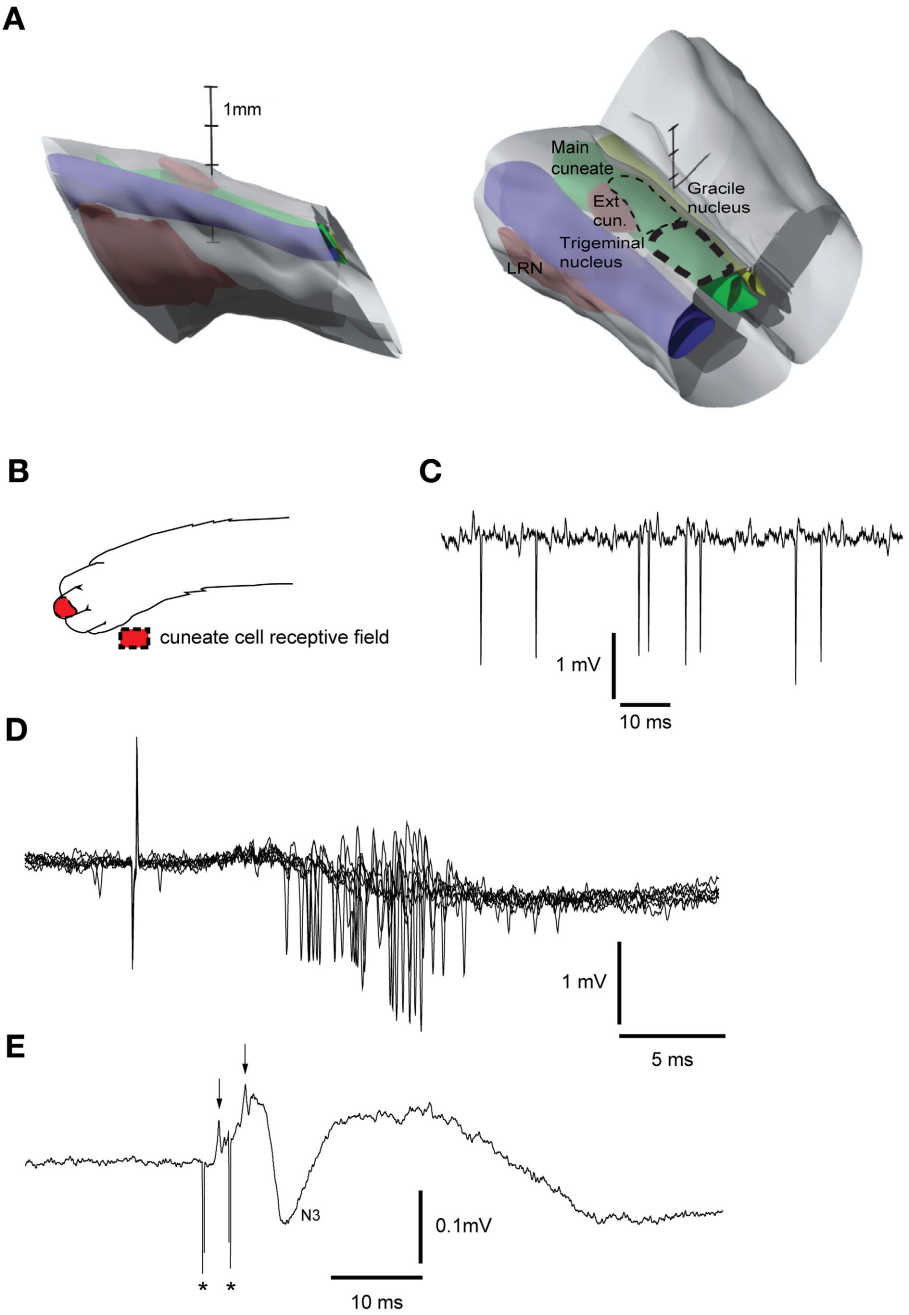
**Reconstruction of brainstem recording/stimulation site and sample recordings. (A)** 3D reconstruction of the brainstem recording/stimulation sites. Thick dashed line represents the primary recording/stimulation area of the main cuneate nucleus. Thin dashed line shows the rostral extent of the recordings. LRN, Lateral reticular nucleus. Vertical scale bar tics: 1 mm. **(B)** Receptive field of cuneate cell recorded in **(C). (C)** Sample recording showing spontaneous cuneate cell activity. **(D)** Cuneate cell responses to electrical skin stimulation (1 pulse, 2 mA). **(E)** Response to electrical stimulation (2 pulses, 20 μA, 333 Hz) in the cuneate nucleus recorded on the surface of the cerebellar cortex. Asterisks indicate shock artifacts. Arrows indicate the afferent nerve volleys elicited by the stimulation. N3 field potential, [an indicator of activity in the parallel fiber synapses of the cerebellar cortex (Bengtsson and Jorntell, 2007)].

A similar tungsten-in-glass microelectrode, exposed tip (10–30 μm), was used to locate the cuneate nucleus (Figure 2A, see below). The electrode was lowered into the brainstem 5–15 mm caudal to the obex, 3 mm lateral to the midline. To evoke a synaptic field and neuronal activity in the cuneate nucleus, the skin of the distal forelimb (Figure 2B) was stimulated electrically through a pair of percutaneous needle electrodes separated by approximately 5 mm (1.2 mA, with single shocks, 0.1 ms long, at 1 Hz). The recording electrode was lowered systematically at different medio-lateral and rostro-caudal positions while recording the spontaneous activity, evoked field potentials and unitary responses to the stimulation throughout the electrode track (Figures 2C,D,E). The electrode was then left in a position where the recorded cells showed characteristic responses of cuneate cells (see Figure 2D). Two loci in the cuneate nucleus, one rostro-ventral and one caudo-ventral, have been reported for the termination of the fibers originating in the red nucleus (McCurdy et al., 1992, 1998). Here we focused on stimulation of the caudo-ventral locus. The cuneate electrode was switched to stimulation mode and subsequently used to condition the pyramidal tract stimulation, by preceding the latter at fixed intervals of 5–300 ms. The stimulation in the cuneate nucleus consisted of 1 or 2 pulses, 0.1 ms wide, with an interstimulus interval (ISI) of 3 ms and a constant current of 5–20 μA. After formaldehyde fixation of the tissue the brainstem was sectioned in 60 μm slices, stained with Cresyl Violet (Svensson et al., 2006) and scanned into an open source 3D-computer program (artofillusion.org) to render a 3D-reconstruction of the brainstem (Figure 2A).

**Figure 3:**
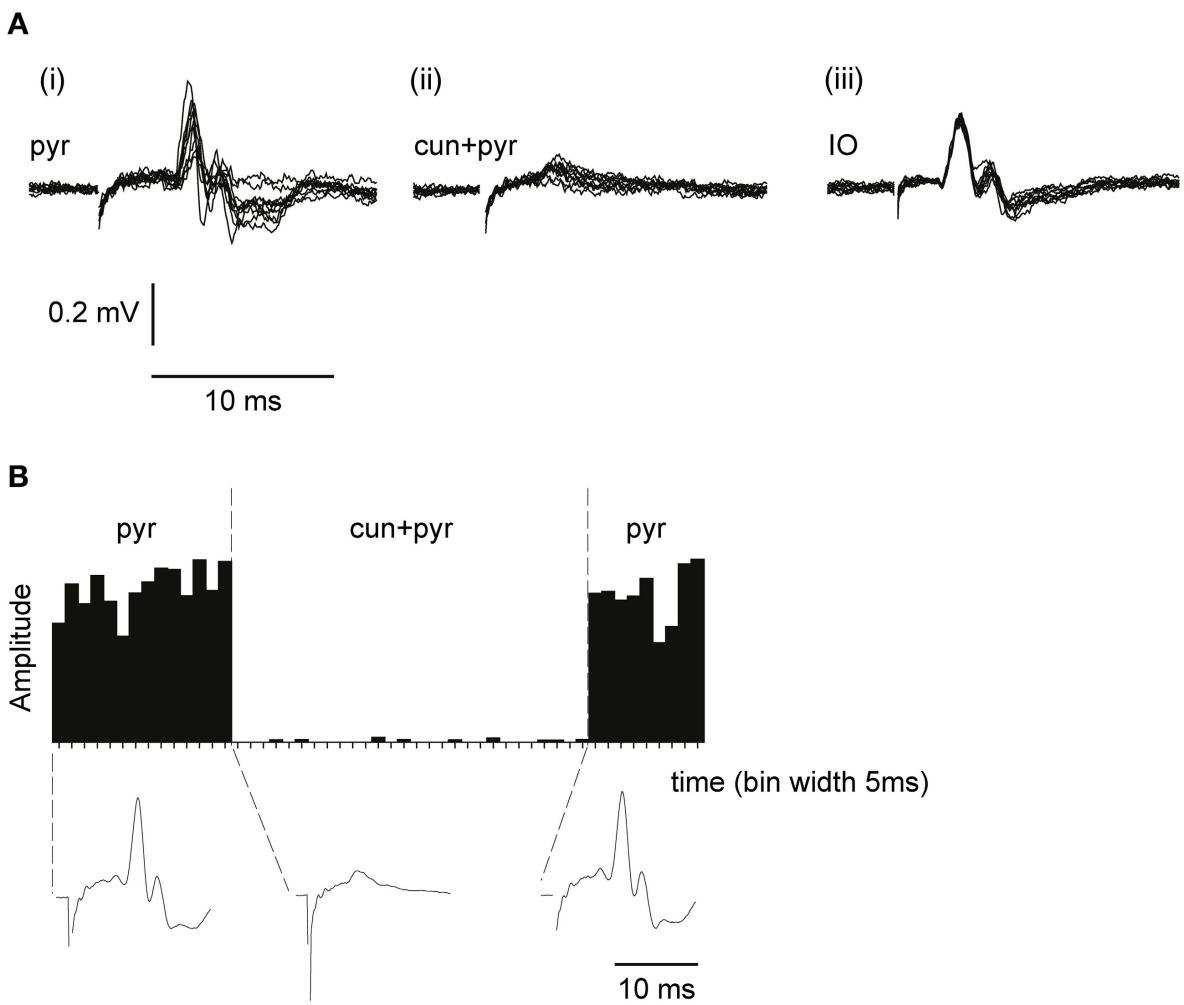
**Climbing fiber field potentials evoked from the pyramidal tract are depressed by a preceding cuneate stimulation. (A)** From left to right, (i) climbing fiber field potential responses in the C3 zone evoked by electrical stimulation in the pyramidal tract (1 pulse, 300 μA, 1 Hz). (ii) Pyramidal tract stimulation preceded (70 ms interstimulus interval) by cuneate stimulation (2 pulses, 20 μA, 1 Hz). (iii) Climbing fiber field response evoked by electrical stimulation in the IO (1 pulse, 30 μA, 1 Hz). **(B)** Sample histogram showing the amplitude of climbing fiber field responses evoked by pyramidal tract stimulation before during and after a preceding (70 ms) cuneate stimulation (2 pulses, 20 μA, 1 Hz). Each bar represents the average of 5 consecutive responses (bin width, 5 s). Below the histogram, average response profiles for the evoked responses.

Evoked synaptic climbing fiber activity was measured from the surface of the cerebellum while stimulating in the contralateral pyramidal tract. The pyramidal stimulation (1 pulse, 100–150 μA, 1 Hz) readily evoked large (300–600 μV) climbing fiber field potentials at a latency of 5–6 ms, sometimes preceded by a smaller mossy fiber field potential (see Figure 3A). Increasing the stimulation frequency beyond 2 Hz gradually reduced the amplitude of the evoked response which is a characteristic of synaptically evoked climbing fiber responses (Armstrong et al., 1968).

### Application of CNQX

In order to test if blocking the excitatory synaptic transmission within the cuneate nucleus affected the suppression of the pyramidal tract response evoked from the cuneate nucleus, we applied small volumes of the ampa-kainate receptor blocker CNQX (6-cyano-7-nitroquinoxaline-2,3-dione, disodium salt) (Disodium salt; Tocris Cookson, Bristol, UK) topically to the surface of the dorsal column nuclei. For this purpose a small piece of filter paper [corresponding to the size of the exposed surface area of the main cuneate nucleus (Figure 2A)] soaked in a solution containing CNQX [5 mM dissolved in phosphate-buffer (0.01 M, pH 7.4)] was placed on the surface of the main cuneate nucleus while we recorded the evoked climbing fiber field potential on surface of the cerebellum (as described above).

The experimental procedures were approved in advance by the local Swedish Animal Research Ethics Committee.

## Results

To test the gating of climbing fiber responses driven by motor command signals, we used the pyramidal tract as a test input. Pyramidal tract stimulation readily evokes climbing fiber responses in the cerebellum (Baker et al., 2001; Pardoe et al., 2004) (Figure 3A). Notably, electrical stimulation within the IO evoked a direct, non-synaptic climbing fiber field potential with a shorter response latency time than the climbing fiber response evoked from the pyramidal tract, consistent with a synaptic activation of inferior olivary neurons from the latter (Figure 3A) The site within the forelimb area of the C3 zone, at which the largest climbing fiber field potential was evoked from the pyramidal tract, was identified. The area of the forelimb from which electrical skin stimulation evoked the largest field potentials at this site was then identified. Subsequently, the cuneate nucleus was identified by stimulating the same forelimb skin site while recording cellular responses in the cuneate nucleus. After having localized a region in the caudal cuneate nucleus activated from this skin site, the electrode was left in position and switched to stimulation mode. We then proceeded by testing if conditioning stimulation in the cuneate nucleus had effects on the climbing fiber field response evoked by the pyramidal tract stimulation.

As is shown in Figures 3A,B, cuneate stimulation could in some cases almost completely block the climbing fiber field potential evoked by pyramidal tract stimulation. At an interval between the cuneate and pyramidal tract stimulations of 70 ms, the pyramidal tract response was substantially depressed (88% ± 4% SEM, *n* = 35, reduction of the evoked climbing fiber response amplitude).

### Latency

**Figure 4:**
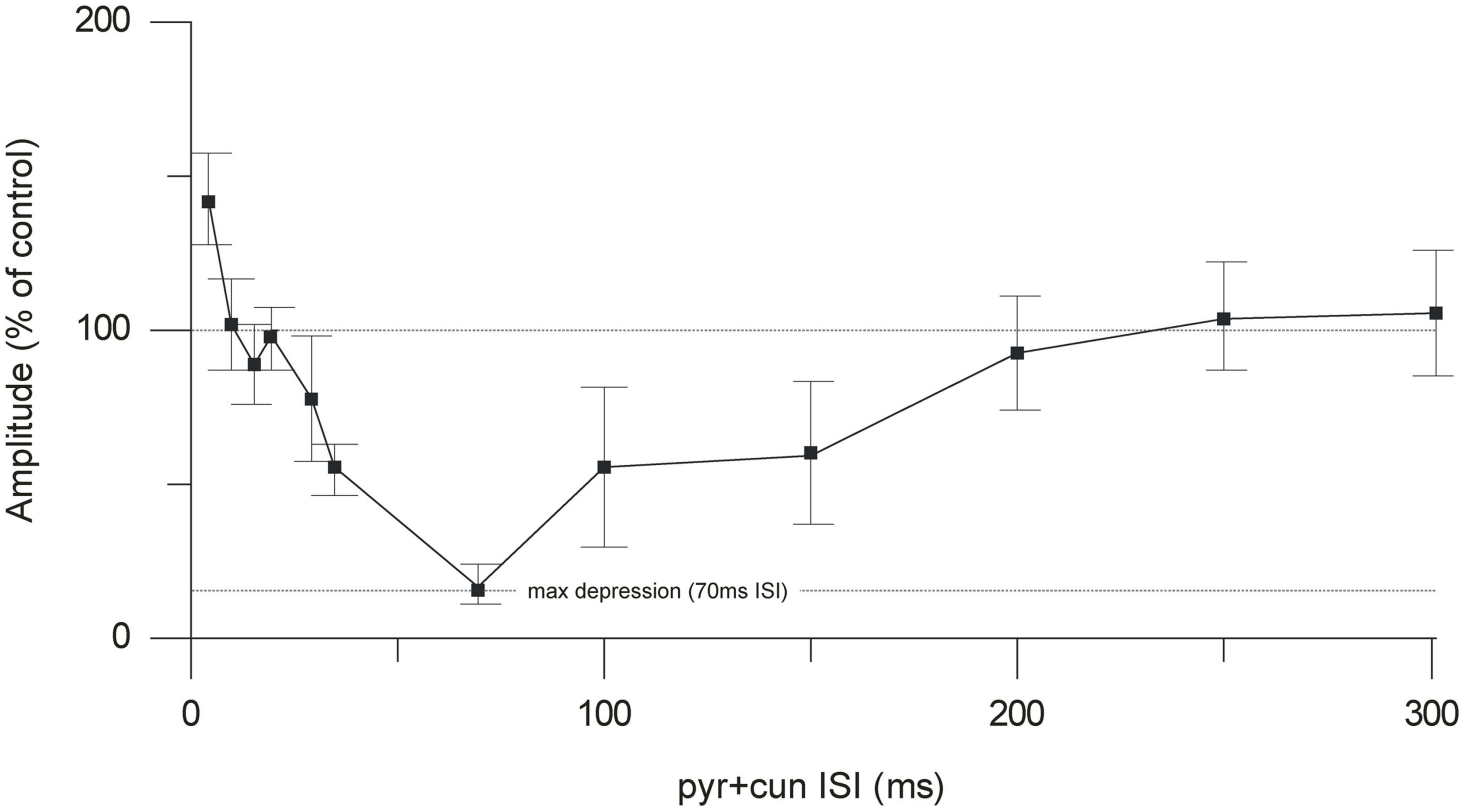
**Time course of the cuneate suppression of climbing fiber field responses evoked from the pyramidal tract.** Average effect (% of control) (mean ± SEM, *n* = 35) of conditioning the evoked climbing fiber field response (70 ms) with cuneate stimulation (2 pulses, 20 μA, 1 Hz).

By changing the interval between the cuneate and pyramidal tract stimulation, we characterized a time profile for the suppression of the climbing fiber field potential. The onset of the suppression was 30–35 ms (Figure 4). Maximal depression of the evoked climbing fiber response amplitude was found at an ISI of 70 ms (88% ± 4% SEM, *n* = 35, reduction of the evoked climbing fiber response amplitude). Interestingly, at intervals shorter than 10 ms, the paired stimulation occasionally (observed in 5 out of 9 cases) resulted in a facilitation of the response evoked from the pyramidal tract (the response amplitude being 140% ± 8% SEM, *n* = 5, of its control value) rather than a suppression. In two animals we tested longer interstimulus intervals (80–300 ms). The results showed a gradual decline in the suppression from 100 to 200 ms interstimulus interval (Figure 3).

### Stimulation strength

**Figure 5:**
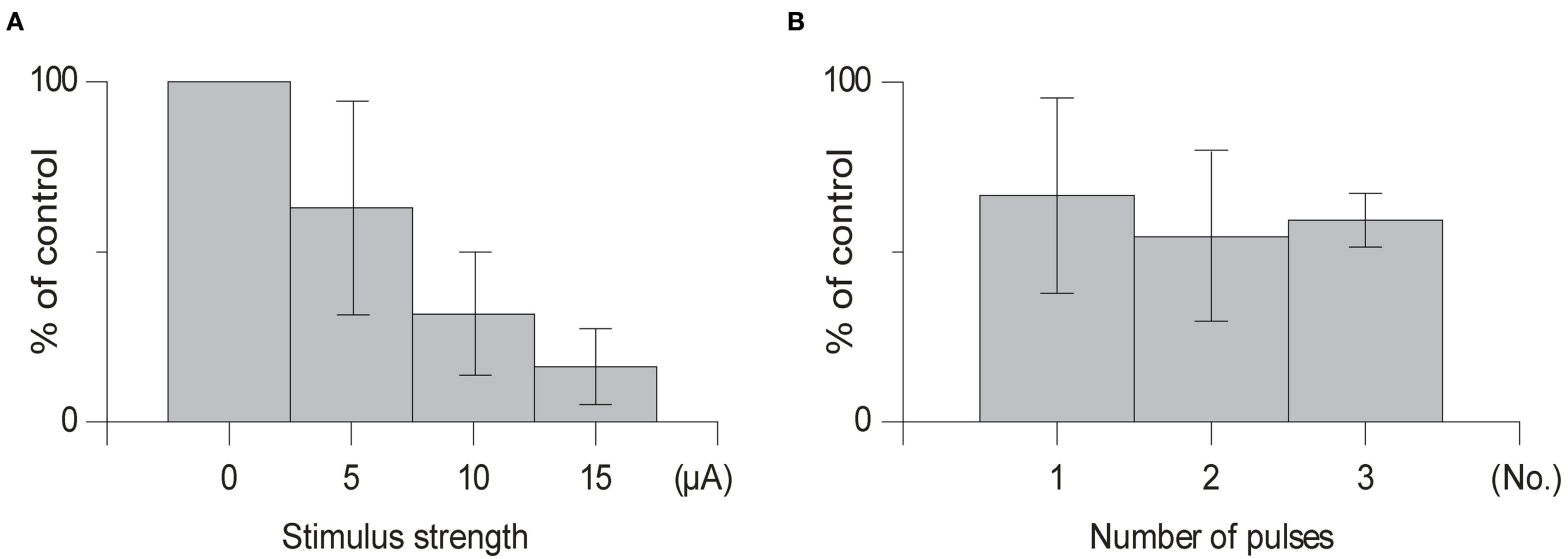
**Dorso-ventral and medio-lateral stimulation profiles. (A)** Depth profile of the effect of the conditioning stimulus in the cuneate on the response amplitude of the climbing fiber field response evoked by stimulation in the pyramidal tract (1 pulse, 150 μA, 1 Hz). The cuneate stimulation preceded the pyramidal tract stimulation by 70 ms (2 pulses, 20 μA, 1 Hz) and was applied at different depths as indicated. **(B)** Depth profile of the conditioning effect in electrode track made 250 μm lateral to the cuneate nucleus. Same stimulation parameters as in **(A)**.

In order to avoid activation of neighboring structures in the brainstem minimum stimulation strengths were used (range 5–20 μA). As can be seen in Figure 5 in some cases the depressive effect occurred already at stimulation strengths as low as 5 μA. However, the effects clearly increased with increased stimulation intensity, when a larger number of cells and fibers in the cuneate nucleus would be expected to be activated.

### Number of pulses

We found that using one pulse was sufficient to suppress olivary transmission and that increasing the number of pulses in the same animal from 1 to 2 or 1 to 3 pulses had little effect (Figure 5) [1 pulse: mean suppression (79% ± 15% SD, *n* = 5); 2 pulses: (66% ± 16% SD, *n* = 4); 3 pulses (59% ± 9% SD, *n* = 4); *t* > 0.14] on the amplitude of the evoked climbing fiber field response. In all cases when testing the effect of the number of pulses the offset of the cuneate stimulation was kept constant.

### Position

**Figure 6:**
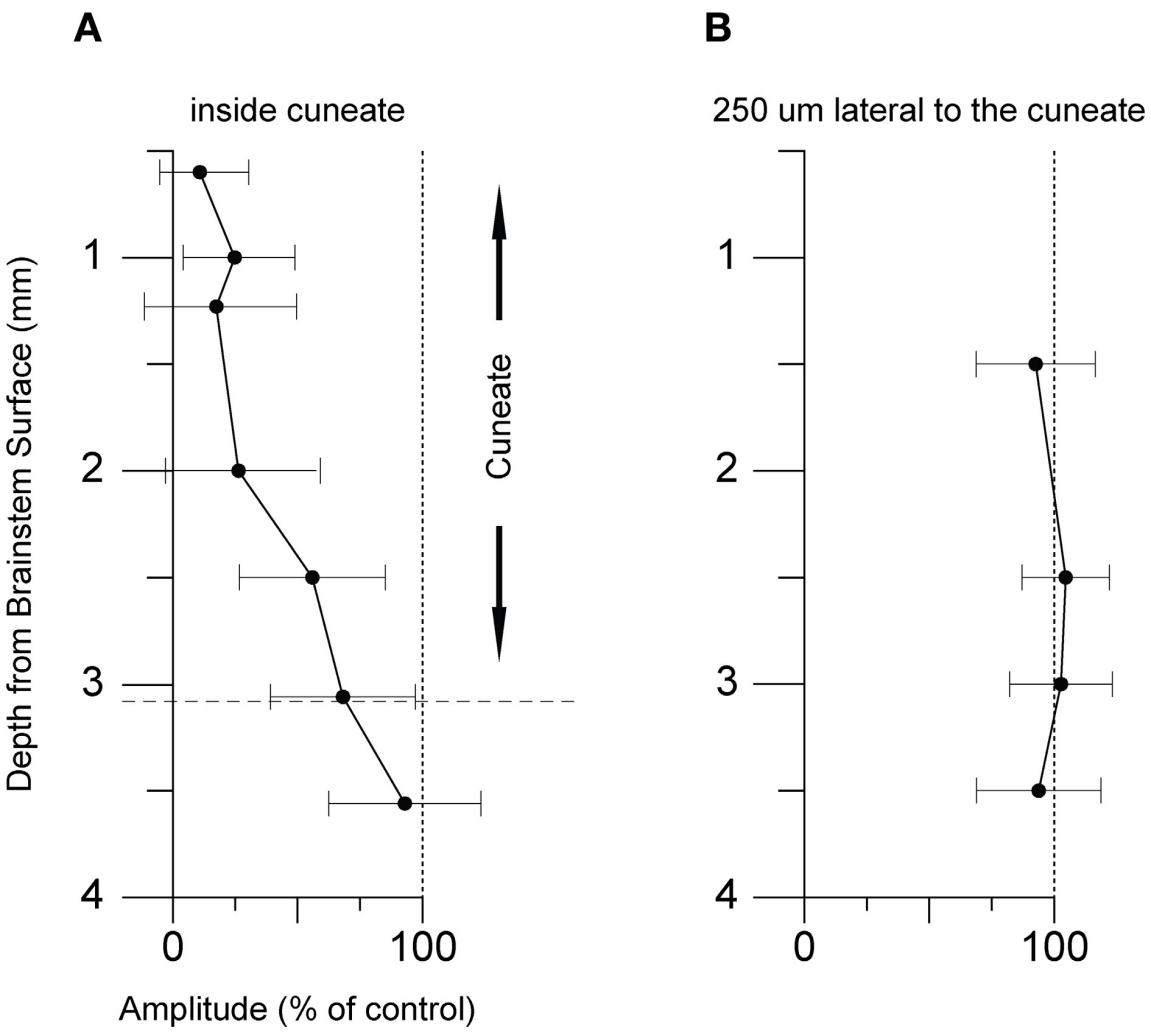
**Effect of varying the stimulation strength and number of stimulation pulses. (A)** Average effect of cuneate stimulation strength on evoked climbing fiber field potential amplitude. Increasing stimulation strength from 5 μA to 15 μA (% of control, mean ± SD, *n* = 5). **(B)** Effect of the number of pulses in the cuneate stimulation train on the evoked climbing fiber field potential amplitude. Increasing number of pulses from 1 to 3 (% of control, mean ± SD, *n* = 5).

Next, we tested whether the inhibitory effects on the pyramidal tract-evoked climbing fiber field responses were confined to the region of the cuneate nucleus. For this purpose we made depth profiles, comparing suppression effects from a number of positions in the caudal cuneate. As the electrode was lowered in the tracks from a dorsal to a ventral position, the depression weakened (Figure 6A). Just ventral to the cuneate nucleus, at 3 mm or more of depth, the depression weakened and eventually ceased. We then proceeded to test the medio-lateral limits of the depression and found that positioning the electrode 250 μm lateral to the cuneate nucleus had no effect on the evoked response (Figure 6B). These findings were hence compatible with that the effect was due to stimulation of elements within the cuneate nucleus.

### Application of CNQX

**Figure 7:**
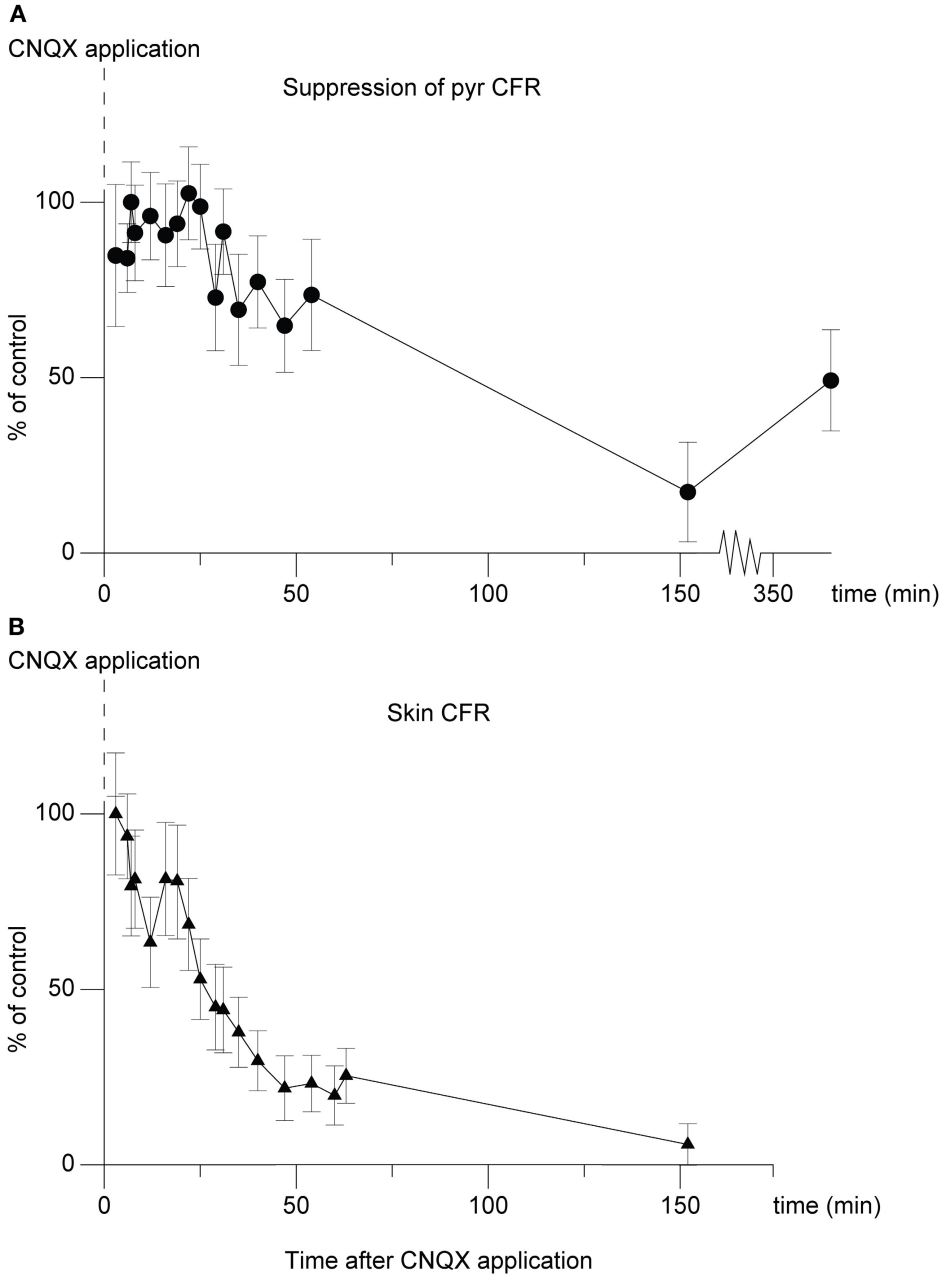
**Effect of CNQX application on the cuneo-olivary suppression and on climbing fiber field responses evoked from the forelimb skin. (A)** Dots (•), shows the effect of CNQX application (5 mM) on the cuneate suppression of pyramidal climbing fiber field responses. Note that in this graph, 100% equals maximal suppressive effect, or control suppression level. The graph displays mean ± SD values for 20 stimulations at each time point displayed. The cuneate stimulation (2 pulses, 20 μA, 1 Hz) preceded the pyramidal climbing fiber field response by 70 ms. **(B)** Triangles (▴), parallel development of the climbing fiber field response evoked by electrical stimulation of the forelimb skin.

In an attempt to further localize the origin of the suppression, in one experiment we applied the non-NMDA ionotropic glutamate receptor antagonist 6-cyano-7-nitroquinoxaline-2,3-dione (CNQX) (Sheardown, 1988), over the region overlying the cuneate nucleus. The effect on the climbing fiber field suppression obtained by paired cuneate and pyramidal tract stimulation was continuously monitored. As can be seen in Figure 7 there was a marked reduction of the suppression. After 54 min the suppression had been reduced by 26% (26% ± 16% SD). After 152 min the effect had been reduced by 83% (83% ± 14% SD). The relatively long time that it took for the effects of CNQX application to develop fully, as well as the almost irreversible nature of the effect (which partly remained 4 h after the filter paper had been removed, 360 min after initial application) are compatible with similar observations made *in vitro* (Andreasen et al., 1989). We also monitored the transmission of synaptic input through the cuneate nucleus by recording the short-latency climbing fiber field response evoked by electrical skin stimulation (1 pulse, 2 mA), a response known to be mediated via the dorsal funiculus and the cuneate nucleus (Ekerot and Larson, 1980, 1982). Roughly in parallel to the developing reduction of the suppression of the pyramidal tract response over time, also the climbing fiber field response evoked from the skin was reduced (peak reduction 94% ± 6% SD at 152 min) (Figure 7). This is in concert with that there are both excitatory and inhibitory connections from the cuneate to the IO (McCurdy et al., 1992, 1998). In contrast, the direct response evoked by the pyramidal tract was unaffected. After 54 min the amplitude of the latter was 109% (109% ± 19% SD) (% of control). After 152 min the amplitude was 115% (115% ± 19% SD) (% of control).

### Nucleo-olivary inhibition

As it is known that output from the deep cerebellar nuclei inhibits olivary transmission (Hesslow, 1986; Bengtsson and Hesslow, 2006; Svensson et al., 2006; Bazzigaluppi et al., 2012) we made two experiments in which we lowered the level of decerebration to a mid-inferior collicular level so that the nucleo-olivary fibers known to run just ventral to the brachium conjunctivum (Legendre and Courville, 1987) were severed. The inhibitory effect observed in the high and low decerebrate preparations could not be separated (*p* = 0.65, student's *t*-test). These findings would suggest that the suppression of the pyramidal tract response was not mediated via the cerebellum.

## Discussion

Here we showed that electrical stimulation within the cuneate nucleus induced a remarkably potent suppression of synaptically evoked climbing fiber field responses. The effect was not obtained lateral or ventral to the cuneate. Blocking excitatory synaptic responses by CNQX applied over the cuneate nucleus essentially eliminated the suppressing effect. This reduction of the suppressing effect occurred in parallel with the development of a reduction of short-latency, skin-evoked climbing fiber field responses known to be transmitted through the cuneate nucleus. All these findings are compatible with the existence of a pathway through the cuneate nucleus being involved in the suppression of climbing fiber excitability. This pathway could be responsible for the inhibition of inferior olivary neuron firing that follows red nucleus stimulation (Weiss et al., 1990; Horn et al., 1998), compatible with the findings that fibers of the RNm terminate within specific regions of the cuneate nucleus and that these parts of the cuneate nucleus projects to the IO. Also pyramidal tract fibers terminate in this part of the cuneate (McCurdy et al., 1992, 1998), meaning that the depression of climbing fiber excitability observed during the initial phase of reaching movements (Horn et al., 1996; Gibson et al., 2002) could potentially involve this pathway. Leicht et al. (1973) found that stimulation of the pericruciate area of the cerebral cortex evoked inhibition of peripherally evoked climbing fiber responses at low thresholds and that the same stimulation evoked excitation of the IO at higher thresholds. An inhibitory pathway through the cuneate which is more easily excited than the pathway of the pyramidal tract to the inferior olivary neuron excitatory synaptic junction is compatible with may explain these findings.

Even single pulse stimulation at very low intensity (5 μA) elicited a strong suppression of the synaptically evoked climbing fiber field responses. McCurdy et al. (1992, 1998) found two ventral termination sites within the cuneate, one rostral, and one caudal, for the rubral fibers. Interestingly, in our case the suppression was most effective from sites located in the dorsal parts of the cuneate. Presumably this is due to the fact that the primary sensory afferent fibers that provide excitation to the cuneate neurons, which run from caudal to rostral just dorsal to the nucleus, branch widely in the rostrocaudal plane (Weinberg et al., 1990) and might hence serve to distribute the effects of the stimulation to a larger population of cuneate neurons. That the main effect of the cuneate nucleus stimulation was due to synaptic excitation of these neurons (the results of the CNQX experiment) is compatible with this interpretation.

The maximum suppression of the pyramidal tract climbing fiber field potential occurred when it was preceded by the cuneate stimulation by 70 ms. This is slightly longer than the response latency times of peak inhibition of inferior olivary neuron responses following red nucleus stimulation (50 ms) (Weiss et al., 1990), although the latency time in this case was calculated from the last pulse in a long train of pulses. Interestingly, these findings roughly match the timing of the inhibition elicited through the nucleo-olivary pathway in the cat (70 ms) and in the ferret (50 ms) (Hesslow, 1986; Svensson et al., 2006). Such long latency times are difficult to explain if one assumes a monosynaptic inhibitory connection. However, a possible explanation proposed is that the GABAergic transmission between the deep cerebellar nuclei and the IO could depend on asynchronous release of GABA, limiting the speed of the synapse (Best and Regehr, 2009).

### Potential mechanisms of the suppression

There are a few not mutually exclusive explanations for the recorded suppression. The first is that there are inhibitory cells projecting to the IO from the cuneate nucleus that are activated by the intra cuneate stimulation. The second is that the suppression occurs as a result of the refractory properties of the IO and as an effect of subthreshold olivary activation. The third is that other pre-olivary regions that have suppressing effects on transmission in the IO were activated.

For the first scenario, the existence of inhibitory (GABAergic) neurons in the cuneate nucleus projecting to the IO have been reported (Isomura and Hamori, 1988; Nelson et al., 1989a,b; Fredette et al., 1992). The cuneate stimulation could naturally result in synaptic excitation of these cells, which would be a straight-forward explanation for our results. Bazzigaluppi et al. (2012) recently showed that there, indeed, is a strong inhibitory effect in the IO cells when the deep cerebellar nuclei are activated and that this effect is dependent on activation of GABA_A_ receptors. Given the similarity of the time course of the effect to our results, the same type of inhibitory mechanisms may also form the substrate for the inhibitory effects of cuneate activation.

For the second scenario, it cannot be excluded that the suppression occurs as an effect of intrinsic refractory properties of the IO. In fact, the facilitation of the pyramidal tract response when the conditioning stimulus occurred at less than 25 ms in advance is compatible with this interpretation. The mechanism would then be that the cuneate stimulation activates excitatory afferents to the rDAO that could cause a subthreshold excitatory response, possibly triggering Ca^2+^ influx in these cells. A Ca^2+^-dependent K^+^ conductance is a prominent feature determining the physiology of the olivary cells (Llinas and Yarom, 1981a,b). The subthreshold Ca^2+^ influx could trigger this conductance, which would result in strong hyperpolarization with a time course that fits our observations. Consecutive synaptic excitations of inferior olivary cells are known to result in a strong suppression of the second response, essentially blocking transmission for intervals shorter than 100–150 ms (Armstrong and Harvey, 1968; Armstrong et al., 1968).

Thirdly, the suppression could have occurred through other brainstem pathways rather than the cuneate nucleus itself. A possible candidate is the relay in the cerebro-olivary pathway across the matrix region (Ackerley et al., 2006), which is located medially and ventrally to the caudal cuneate nucleus. However, this candidate is made less likely since the stimulating current required to evoke the suppression from the dorsal part of the cuneate was extremely low, the primary afferent fibers in this region run rostro-caudally rather than medio-laterally and are not known to make synapses with neurons in the matrix region. By lowering the decerebration level to a mid-collicular position we could exclude that the suppression originated in the deep cerebellar nuclei. This would for example, rule out that the effects of the stimulation resulted from synaptic excitation of cells in the lateral reticular nucleus, which would synaptically excite the deep cerebellar nuclear cells (Wu et al., 1999; Shinoda et al., 2000) and inhibit the IO via the nucleo-olivary cells (Bengtsson and Hesslow, 2006).

The present study illustrates that weak electrical stimulation within the cuneate nucleus, in particular its dorsal part, elicits a powerful suppression of synaptically evoked climbing fiber field responses. Combined with the results from numerous anatomical studies, we conclude that the cuneate nucleus is a possible candidate pathway involved in the suppression of inferior olivary excitability observed from the onset of reaching movements (Horn et al., 1996; Gibson et al., 2002).

In summary, inhibition of the IO seems to be an important feature, this especially so during active movement (Horn et al., 1996; Apps, 1999; Apps and Lee, 1999; Gibson et al., 2002). So far, at least two separate but parallel inhibitory pathways that are active during movement have been identified. The first, the nucleo-olivary pathway known to exert a powerful inhibition of the IO (Hesslow, 1986; Svensson et al., 2006; Bazzigaluppi et al., 2012) and to be active during the expression of conditioned reflex movements (Hesslow and Ivarsson, 1996). The second, the cuneo-olivary pathway, which most likely is activated by a number of different sources acting on the cuneate, like the red nucleus (McCurdy et al., 1992, 1998) and the cerebral cortex (Leicht et al., 1973), during movement. The common feature of these pathways is that they are all activate during movement and thus probably at least partly responsible for the lack of relationship between olivary discharge and movement. However, further studies are needed to explore if there are yet other pathways that can inhibit olivary transmission during active movement.

### Conflict of interest statement

The authors declare that the research was conducted in the absence of any commercial or financial relationships that could be construed as a potential conflict of interest.
